# Estimating incidence trends in regular heroin use in 26 regions of Switzerland using methadone treatment data

**DOI:** 10.1186/1747-597X-4-14

**Published:** 2009-06-12

**Authors:** Carlos Nordt, Karin Landolt, Rudolf Stohler

**Affiliations:** 1 Research Group on Substance Use Disorders, Psychiatric University Hospital, Zurich, Switzerland

## Abstract

**Background:**

Regional incidence trends in regular heroin use are important for assessing the effectiveness of drug policies and for forecasting potential future epidemics.

**Methods:**

To estimate incidence trends we applied both the more traditional Reporting Delay Adjustment (RDA) method as well as the new and less data demanding General Inclusion Function (GIF) method. The latter describes the probability of an individual being in substitution treatment depending on time since the onset of heroin use. Data on year of birth, age at first regular heroin use and date of admission to and cessation of substitution treatment was available from 1997 to 2006 for 11 of the 26 regions (cantons) of Switzerland. For the remaining cantons, we used the number of patients in 5-year age group categories published in annual statistics between 1999 and 2006.

**Results:**

Application of the RDA and GIF methods on data from the whole of Switzerland produced equivalent incidence trends. The GIF method revealed similar incidence trends in all of the Swiss cantons. Imputing a constant age of onset of 21 years resulted in almost equal trends to those obtained when real age of onset was used. The cantonal incidence estimates revealed that in the mid 80s there were high incidence rates in various regions distributed throughout all of the linguistic areas in Switzerland. During the following years these regional differences disappeared and the incidence of regular heroin use stabilized at a low level throughout the country.

**Conclusion:**

It has been demonstrated that even with incomplete data the GIF method allows to calculate accurate regional incidence trends.

## Background

A lack of information about trends in the incidence of regular heroin use hinders effective drug policy and public health action. Yet incidence trends are still unknown almost everywhere in the world. Various estimation methods have been developed to estimate the incidence of heroin use [1-4], however they have requirements, like long-term treatment data or reliable drug mortality statistics, which are rarely available. We have recently reported estimates of incidence and prevalence trends in regular heroin use in Zurich, Switzerland [5]. These estimates were produced using the Reporting Delay Adjustment (RDA) method and were based on data from a long-term case register that covered all methadone treatments for more than a decade. The RDA method led to prevalence estimates that were in good accord with prevalence estimates generated by other approaches. We have also developed a simpler procedure to estimate the incidence of regular heroin use, called the General Inclusion Function (GIF) method, which only requires methadone treatment data from a single day [6]. On the premise that heroin dependence is usually a chronic condition, we have hypothesized that if governmental regulations do not restrict access the probability of an individual being in substitution treatment depends largely upon time since onset of regular heroin use. The GIF approach led to reasonably good incidence estimates in the canton of Zurich, despite the presence of open drug scenes and irrespective of whether onset of regular heroin use occurred during an early or a late phase of the 'heroin epidemic'.

To explore if the GIF method yields plausible incidence estimates in other regions, we decided to apply it to other Swiss cantons. There are several advantages of using other areas in Switzerland to test the GIF method. First, even though incidence trends in regular heroin use are unknown in almost all regions, drug mortality trends and the stable number of patients in substitution treatment suggest that prevalence trends are similar throughout Switzerland. Second, each of the 26 cantons in Switzerland is individually responsible for treatment provision and data collection. Third, the size of the population of regular heroin users differs vastly between cantons. For example, in 2003, the annual number of individuals in methadone treatment in various cantons ranged from 3 to 3,592 [7]. Therefore, by using separate datasets that should yield similar incidence trends in each canton, we can determine the smallest area to which the GIF method can be applied.

Unfortunately, not every Swiss canton has a methadone treatment register that includes data on the year of first regular heroin use. Since heroin use usually occurs early in life [1], when data on the age of heroin onset are missing, an estimate of the number of affected individuals in each birth cohort may be used as an approximation for incidence estimates. A benefit of using data collected on the year of birth of patients in substitution treatment is that it probably contains fewer errors than age at first regular heroin use. If we find similar incidence estimates in several adjacent regions and similarly affected birth cohorts, estimates on birth cohorts in the remaining regions would be sufficient to draw a rough picture on how a 'heroin wave' has spread over the whole area.

Thus, the aims of this paper are to: (i) estimate the incidence of regular heroin use for the whole of Switzerland using the Reporting Delay Adjustment (RDA) method; (ii) ascertain a General Inclusion Function (GIF) for the whole of Switzerland by using the annual number of patients in substitution treatment; (iii) apply the GIF function to treatment data from different calendar years, using data on 'year of first heroin use' as well as 'year of birth'; and (iv) examine if assuming a constant age of onset leads to acceptable incidence trend approximations in all Swiss cantons.

## Methods

### Study area

Open drug scenes started to develop in Switzerland in the early 1980s, mainly in the city of Zurich, but also in other German speaking parts of Switzerland. The French and Italian speaking parts of Switzerland were not affected. Thus, linguistic regions may differ concerning the development of the heroin wave. In February 1995, the last open drug scenes in Zurich, Solothurn, and Olten were closed; those in Bern, Basel-Stadt, and St. Gallen had been closed earlier [8].

### Methadone treatment registers

The Swiss law on narcotics requires that treatment providers obtain permission from cantonal health authorities to prescribe opioids to people dependent on heroin. The law also requires a register of substitution treatments provided to opioid dependent persons. Each of the 26 Swiss cantons is responsible for methadone treatment provision and data collection. The Swiss Federal Office of Public Health has collated and published annual statistics from all cantons since 1999 [7]. Since 2002, it has provided a computer program to all interested cantons by which more detailed treatment data can be collected and subsequently accessed. We obtained most of the register data via the Swiss Federal Office of Public Health. Variables included a personal identification number, date of birth, age at first regular heroin use, date of admission to and cessation of each treatment, and date of the first lifetime treatment episode. Although the same database was used, most, but not all, cantons supplied time-ranges of permissions to treat a specific patient, lasting several months to a year. Moreover, cantons collected age at first regular heroin use to varying degrees.

Data from this database and other datasets were analyzed, if cantonal health authorities agreed to participate in the study. The following 11 cantons were enrolled: Bern, Fribourg, Genève, Graubünden, Neuchâtel, Schaffhausen, Solothurn, St. Gallen, Ticino, Vaud, Zug, and Zurich. Additionally, we obtained the exact year of birth of all patients in treatment on a specific day for 2 cantons (Aargau, 28^th ^of January 2008, n = 812; Solothurn, 17^th ^of January 2008, n = 621). We did not approach most of the remaining 13 cantons because they had limited sample sizes, generally having less than 100 individuals in treatment. Data from the 11 cantons fully included in this part of the study represented about 70% of the Swiss population in methadone treatment. These 11 cantons were spread throughout different linguistic areas and included both urban and rural parts of Switzerland.

### Data preparation

We examined the data for consistency. If heroin onset was recorded to be before the age of 12, if onset was not recorded to be in the year of first substitution treatment or before, or if discrepancies between a patient's year of onset recorded in different treatment episodes were greater than 3 years, the year of onset from this treatment episode was considered as an error in the data and thus deleted. If there were discrepancies in the year of onset for an individual patient of 3 years or less, a mean value was calculated and rounded accordingly. For the remaining patients, we assumed that the missing data did not strongly deviate from the data obtained for the 14,396 (53%) patients with a known year of first regular heroin use. For the RDA method we used all available data from the 11 cantons included in this study. For the GIF method we only used data from those calendar years that had similar numbers of admissions and cessations. This ensured that in the analyses, we only included data from years in which a register had been fully operational.

### Calculating incidence estimates using the RDA method

The RDA method aims to estimate and to correct for those heroin users from each onset cohort, who have not yet shown up in opioid substitution treatment. It assumes a stable probability distribution that describes the time between onset of heroin use and first treatment. If we assume that almost every regular heroin user enters his first substitution treatment (if ever) within 10 years, a treatment register of ten years would be sufficient to estimate a full probability distribution of that 'delay' or 'lag'. To estimate the lag time period between first regular heroin use and first substitution treatment we applied the method proposed by Hickman and colleagues [2]. To avoid bias due to restricted availability of treatment slots or missing data before 1997, we only included individuals (n = 2,709), who had begun using heroin regularly between 1997 and 2006. We estimated the conditional lag distribution of this 10-year period with a general linear model using five parameters (a linear and a quadratic term of the lag time and dummy variables for the three shortest lag time periods), as in a former publication [5].

The RDA method underestimates incidence because it does not account for those heroin users, who do not show up in treatment. Thus, we applied a cessation correction of 4% per year, based on the assumption that each heroin user observed in treatment is representative of all those who had died or stopped heroin use before having had the possibility to enter treatment. For example, if a total of 44 individuals, who entered treatment in 1997, reported initiating regular heroin use in 1977, this suggests that 100 individuals had started regularly using heroin in 1977 (= 0.96^20 years^).

### Estimating the annual probability of being in substitution treatment (GIF)

We divided the annual number of individuals in substitution treatment by the RDA incidence estimate for each calendar year between 2001 and 2006 (n = 12,846 to n = 13,336). Since the data revealed a preference for reporting even years of regular heroin onset, we computed a three-period moving average using the former, the current, and the following values. The resulting treatment probabilities were plotted by time since onset of regular heroin use.

### Applying the GIF to year of first regular heroin use

To estimate incidence for each calendar year separately, we applied an approximation formula for the General Inclusion Function. This formula describes the probability of being in treatment at least 1 day during a year (*P*_annum_), depending on time since onset (in years):



The components of this approximation formula can be interpreted as follows: 0.7 is a linear scale factor and sets the peak to 0.457; the term (1 – (time-0.2)^-1^) describes the steep rise in probability during the first years; after some decades the decline approximates 4% per year (0.96^(time-0.2)^).

### Estimating the number of affected individuals in each birth cohort

The mean age at first regular heroin use was approximately 21 years in all cantons over all treatment years. Thus, we estimated the number of affected individuals in each birth cohort in cantons providing detailed data by supposing a constant age of onset of 21 years and applying the approximation formula for the General Inclusion Function.

### Approximating incidence, using estimates of the number of affected individuals in each birth cohort

Shifting the time scale by 21 years – the mean age of onset of regular heroin use – allowed comparisons to be made between incidence estimates based on age of onset data and those based on birth cohort data. Theoretically, the narrower the age of onset distribution, the more similar the two forms of incidence estimates.

### Estimating the number of affected individuals in each birth cohort, using data in age groups, published in annual treatment statistics

Between 1999 and 2006, 24 out of 26 cantons published the number of patients in treatment by 5-year age group categories over at least several years [7]. To estimate how many individuals of a specific age were in treatment per year, we divided the published number of patients within each age group by 5. We assumed that the resulting number was the best estimate for the size of the specific group, whose age corresponds to the mean age of the category. We obtained a value for the number of patients aged 22, 27, 32 and 37 for each treatment year between 1999 and 2006. This procedure led to the number of individuals in treatment for each birth cohort between 1962 and 1984. When we obtained more than one number for a specific birth cohort, we used the value from the most recent treatment year. Although only 7 out of 24 cantons had published data on age groups for each year between 1999 and 2006, 15 out of 24 cantons had data available from 2002 to 2005. For the canton of Basel-Stadt we had to rely on a publication that only provided 10-year age group categories [9]. Therefore, for this canton we modified our procedure accordingly.

In cantons for which we could recalculate the numbers of patients in treatment using treatment case register data, we found some differences in the size of age groups. Since we were interested in how well the procedure using 5-year age group categories fitted estimates using numbers of patient of each birth cohort from treatment case registers, we used our own calculated annual numbers of 5-year age group categories.

## Results

For 11 cantons we had complete methadone treatment register data from 1998 to 2006 (Table 1). The mean annual number of patients treated during the respective years varied from 3,701 in the canton of Zurich to 79 in the canton of Zug. The mean age was approximately 35 years and the mean age of onset of regular heroin use was approximately 21 years. The proportion of cases with known age of onset ranged from 6% to 90% between cantons. In most cantons and during all treatment years the mean age of patients with unknown age of onset was about 3 years higher than the age of patients with known age of onset.

**Table 1:** Methadone treatment case-register data.

	Treatment years included	Number of patients (mean)	Age (mean)	Age at first regular heroin use (mean)	Proportion with known onset (mean)
Zurich	1998–2006	3,701	35.4	21.5	76%
Bern	1999–2005	2,650	34.8	20.9	50%
Genève	1999–2006	1,581	37.3	20.1	16%
Vaud	2001–2006	1,684	36.0	20.8	36%
Ticino	1998–2006	987	35.5	20.8	90%
St. Gallen	2001–2007	910	35.2	22.9	31%
Neuchâtel	2000–2006	689	36.1	21.1	32%
Fribourg	1999–2005	496	33.4	20.2	51%
Graubünden	1998–2006	267	34.3	19.7	6%
Schaffhausen	2000–2006	140	33.9	19.2	28%
Zug	2006	79	36.2	20.8	72%

Methadone treatment case-registers data from 11 cantons of Switzerland, 1998–2008.

**Figure 1:**
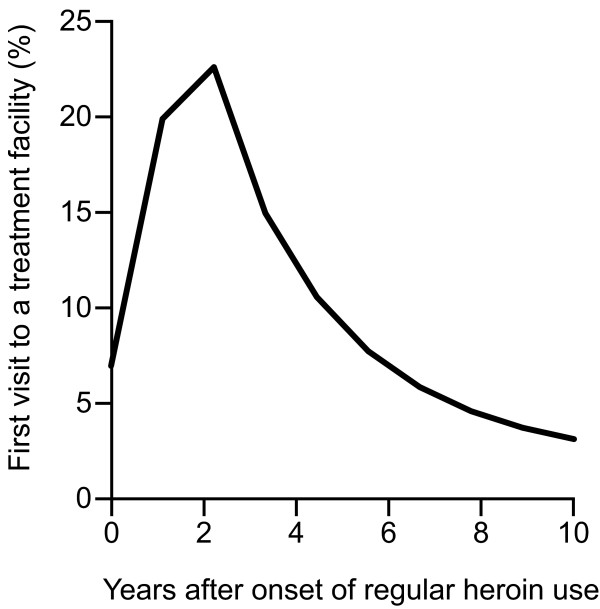
**Lag-time distribution (RDA)**. Joint lag-time distribution between onset of regular heroin use and first visit to a substitution treatment facility in the 11 cantons of Switzerland that provided year of onset, 1997–2006.

Data from the case registers of all 11 participating cantons showed that in total 27,047 patients had entered substitution treatment until the end of 2006. The RDA method revealed that heroin users entered their first substitution treatment soon after onset, i.e. 22.6% entered two years after onset (Figure 1), if they did so within the period of 10 years that was covered by the data. Thus, summing the lag-time distribution up to year two, we see that one half of heroin users (49.5%) entered substitution treatment for the first time within two years of onset.

**Figure 2:**
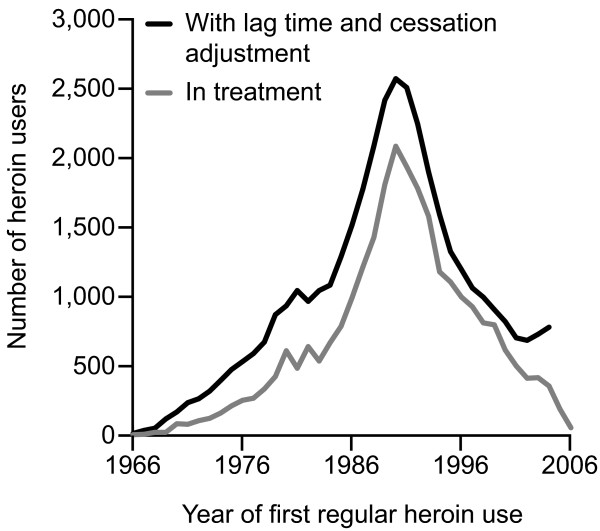
**Incidence by the RDA method**. Joint incidence of regular heroin use in the 11 cantons of Switzerland that provided year of onset estimated by the RDA method, 1966–2006.

Adjustment of the observed incidence number by the lag-time distribution and cessation correction only affected the overall shape of the heroin incidence curve to a small extent (Figure 2). The adjusted incidence curve indicates that heroin use first began in 1966 with 15 individuals. Following this there was a steep rise in heroin incidence, which peaked in 1990 with 2,572 new users, and then a steep decline to 686 users in 2002. Incidence levels have remained stable since then.

**Figure 3:**
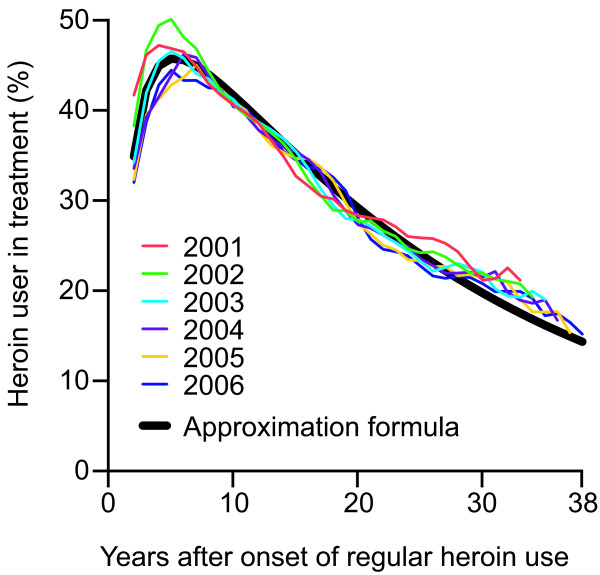
**Annual treatment probability by time since onset (GIF)**. Joint annual probability of being in treatment for at least one day among all regular heroin users of the 11 cantons of Switzerland that provided year of onset, 2001–2006.

Applying the GIF approach, a steep increase in the proportion of regular heroin users in treatment is observed during the first years after onset (Figure 3). Between 2001 and 2006, 5 years after onset of regular heroin use between 42.8% and 50.2% of users were in substitution treatment for at least 1 day. The proportion of heroin users in treatment declined slowly to about 21% three decades after onset.

**Figure 4:**
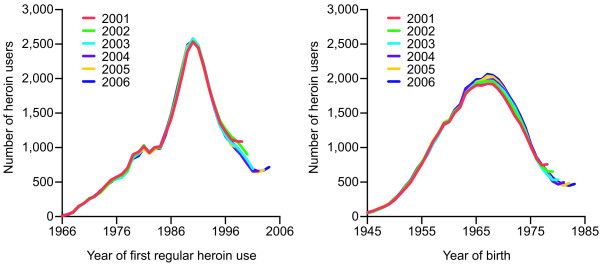
**Incidence and affected birth cohorts by the GIF method**. Joint incidence of regular heroin use and affected birth cohorts in the 11 cantons of Switzerland that provided year of onset; annual estimates by the GIF method using number of patients, 2001–2006.

The left panel in figure 4 displays the resulting incidence estimates when the GIF approximation formula is applied to the joint dataset of the 11 cantons. It shows that this formula yields similar results using the annual number of patients from 2001 to 2006, respectively. The right panel in figure 4 shows the results of the application of the approximation formula on year of birth when imputing a constant age of onset of 21 years instead of real age of onset. Again, the approximation formula yields similar results using the annual number of patients from 2001 to 2006, respectively. Results indicate that with approximately 2,000 individuals in each cohort, the birth cohorts between 1965 and 1969 were most affected by regular heroin use.

**Figure 5:**
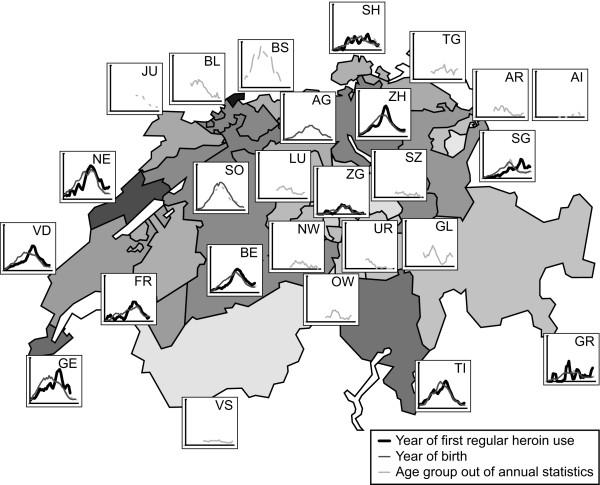
**Standardized incidence estimates by the GIF method**. Standardized estimates of incidence of regular heroin use per 1,000 population and its approximation by using year of birth or 5-year age group categories from annual tables of all 26 cantons of Switzerland, 1970–2005. Gaps in lines derive from missing annual tables in the National Statistic of Methadone Treatment between 1999 and 2006 [7].

The annual incidence estimates from all 11 cantons were very similar, especially within those cantons with a high number of methadone treatments and a high proportion of known years of onset. Figure 5 shows the time trends of standardized incidence estimates in all cantons of Switzerland. The bold line indicates the best incidence estimates using year of onset. Incidence trends were similar between almost all cantons with a clear peak in the very early 90s, except for the canton of St. Gallen (SG). The 2 thinner lines depict the incidence trends, calculated by year of birth, shifted by 21 years. Even though the overall trend of all 3 lines is similar within cantons, the trends calculated by year of birth generally show a less pronounced peak.

**Figure 6:**
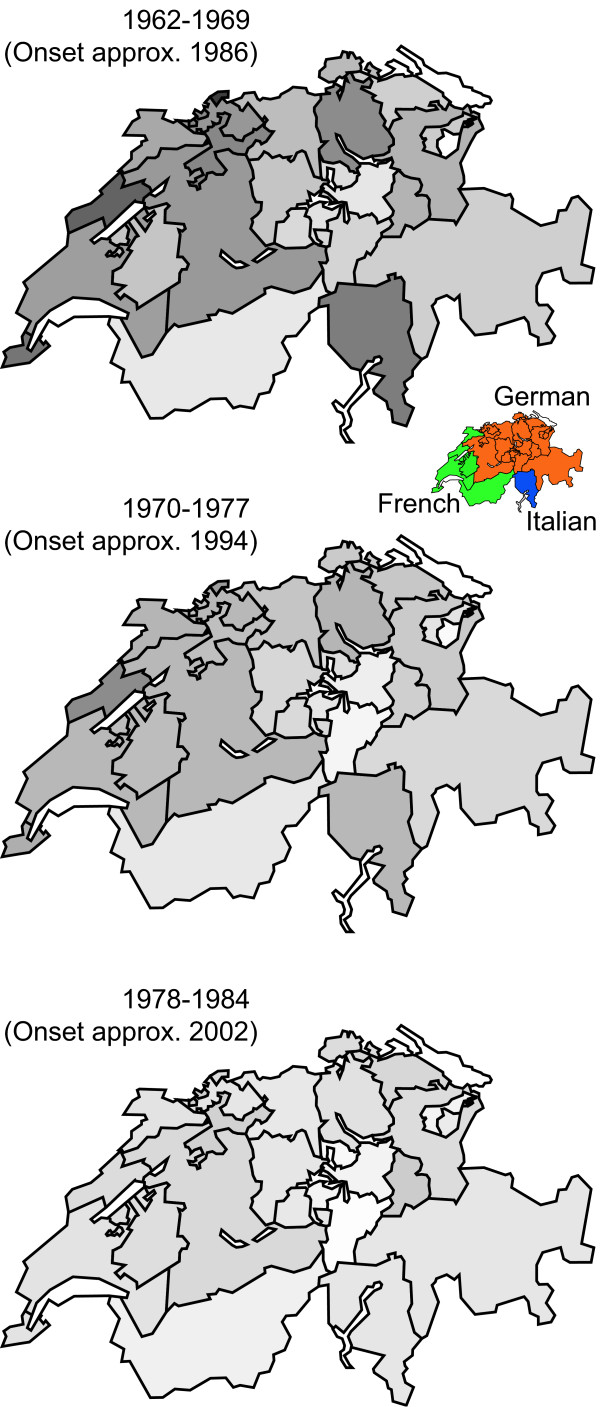
**Evolution of the 'heroin epidemic'**. Evolution of the 'heroin epidemic' in 3 phases using approximated incidence trends of regular heroin use per 1,000 population during 2 decades in all 26 cantons of Switzerland. The darker the area the higher the affected birth cohorts in the respective canton, using 5-year age group categories from annual statistic tables between 1999 and 2006.

Figure 6 displays approximated incidence trends over time in each canton of Switzerland. Darker colors represent higher incidence rates. Using estimates of the number of affected individuals born between 1962 and 1969 and initiating regular heroin use around 1986, we found 5 areas – each comprising 1 or more cantons – with higher incidence rates. These regions were spread over all linguistic areas of Switzerland. Over the years, differences in incidence rates between cantons disappeared and heroin incidence now seems to have stabilized at a comparatively low and equal level throughout Switzerland.

## Discussion

This study has shown that the GIF method allows us to estimate the incidence of regular heroin use even in small areas or with incomplete data. By joining long-term treatment register data from 11 cantons, we found that in Switzerland one half of regular heroin users had entered substitution treatment for the first time within two years of onset. Since the 11 cantons included in the study provide approximately 70% of all opioid substitution treatments in Switzerland, we estimate that in the whole of Switzerland, the incidence of regular heroin use peaked with about 3,675 new users in 1990, and that about 1,000 individuals began regularly using heroin per year from 2001 onwards.

In all cantons, the mean age of onset of regular heroin use was about 21. Therefore, supposing a constant age of onset of 21 years and applying the GIF method we estimate that for the whole of Switzerland the birth cohorts between 1965 and 1968 were most affected with approximately 2,800 (≈2,000/0.7) individuals initiating regular heroin use.

Using year of birth and assuming onset of regular heroin use at the age of 21 years led to reasonably good approximations of incidence estimates obtained by real age of onset. If we suppose that any short peak of heroin incidence will affect young people at around the age of 20, it follows that birth cohort incidence curves will be less steep and smoother than year of onset incidence curves. The results from different Swiss cantons showed some support for this assumption, with birth cohort trend estimates peaking a few years earlier at lower levels.

Given the relatively high proportion of missing data on age of onset, the variation in the proportion of missing data between cantons and the fact that the mean age of those patients with missing data on age of onset was about 3 years higher than that of patients with available data, we must be careful when comparing incidence trends between cantons. If data concerning age of onset is of poor quality, using year of birth and calculating incidence by birth cohort, may lead to better estimates. Among our incidence estimates, the canton of St. Gallen is a good example, where a rather pronounced difference between birth cohort estimates and year of onset estimates point to possible weaknesses in the quality of data on age of onset. However, even if birth cohort estimates are more robust, they do not necessarily correspond fully to onset incidence trends.

In almost all cantons with elevated incidence levels, the birth cohorts of the late 60s and the early 70s were most affected by heroin use. Due to the small size of some cantons and some data of debatable quality, we think that grouping estimates into 7-year periods is more appropriate than speculating about the 'irregularities' of estimated trends. Despite this low time resolution, our representation of the spread of the 'heroin epidemic' in its spatial and temporal dimensions is still meaningful. It clearly reveals that there were several regions with higher incidence rates during the 80s. These 'centers' are distributed throughout all linguistic regions of Switzerland. This might be surprising since open drug scenes have only been known to exist in the German part of Switzerland. Thus, we have no indication of whether the 'heroin wave' begun in the eastern or in the northern part of Switzerland, nor whether it spread from a centre – for example, from Zurich, with its more prominent open drug scenes. Over the last 2 decades, the former spatial differences seem to have disappeared and the incidence of regular heroin use has stabilized at a comparatively low level throughout Switzerland.

Apart from the afore-mentioned limitations, our approach should not be understood as providing the 'best possible estimate' of the incidence of regular heroin use for each canton of Switzerland. In this paper, we neglected the number of opioid users in heroin-assisted treatment programs. This form of treatment exists in about half of all cantons in Switzerland, most often in the German part [10]. But as the proportion of opioid users in heroin-assisted treatment amounts only to about 10% of those in methadone treatment, this would not lead to substantially different results. A probably greater influence on estimates in specific cantons is the implicit assumption that there was no long-term drift of heroin users towards more urban regions. Short-term drifts are less probable because of the activities of cities with open drug scenes that aimed to 'repatriate' users of illegal drugs to their former living community (see for example a project established in 1993 in the city of Zurich [11]). However, a long-term shift may have led to an underestimation of incidence trends in smaller cantons, where trends are difficult to estimate due to small numbers. Taken together, we recommend that incidence estimates for each calendar year should not be made for areas with less than 300 patients in substitution treatment. For greater time-frames even about 100 patients in substitution treatment will be sufficient.

## Conclusion

Using a simple method to estimate the incidence of heroin use, we were able to show that it is also possible to estimate incidence trends in relatively small areas and with incomplete data.

As to the spread of the heroin wave in Switzerland, in all cantons incidence peaked at the beginning of the 90s. Those cantons with higher incidence rates during the 80s were not necessarily those cantons where open drug scenes were present. Over the last 2 decades the former spatial differences in Switzerland seem to have disappeared, and the incidence of regular heroin use has stabilized at a comparatively low level throughout Switzerland.

## Competing interests

The authors declare that they have no competing interests.

## Authors' contributions

CN was the main contributor to the design of the study and did the statistical analyses. All authors participated in the execution of the study and the writing of the manuscript. All authors read and approved the final manuscript.
